# Effects of Omalizumab on FcεRI and IgE Expression in Lesional Skin of Bullous Pemphigoid

**DOI:** 10.3389/fimmu.2019.01919

**Published:** 2019-08-14

**Authors:** S. Morteza Seyed Jafari, Karolina Gadaldi, Laurence Feldmeyer, Nikhil Yawalkar, Luca Borradori, Christoph Schlapbach

**Affiliations:** Department of Dermatology, Inselspital-Bern University Hospital, University of Bern, Bern, Switzerland

**Keywords:** bullous pemphigoid, FcεRI, IgE, omalizumab, skin

## Abstract

Recent studies suggest an important role of immunoglobulin E (IgE) as an alternative pathogenic pathway in the development of bullous pemphigoid (BP), as the most frequent subepidermal blistering disease of the skin Use of IgE targeted therapies, such as omalizumab, has been shown promising in recent studies. The aim of this study was to assess the effect of omalizumab on FcεRI and IgE expression on circulating basophils and on lesional intradermal cells in BP to generate insight into the immunological effects of omalizumab in BP. We report two cases of BP patients treated with omalizumab. Efficacy of treatment was assessed clinically 4 months after initiation of the therapy. Lesional and non-lesional skin biopsies where taken before and 4 weeks after initiation of omalizumab therapy. In addition, FcεRI expression on circulating cells and IgE levels in serum and in the skin samples, as well as anti-BP180 and anti-BP230 in serum and eosinophils and basophils counts in blood were assessed before and during treatment. Both patients showed a marked improvement after 4 months, with no adverse effects. Down-regulation of FcεRI, IgE in lesional skin and on circulating basophils were observed in parallel with clinical improvement. The current case study supports the role of omalizumab in the treatment of a subset of BP patients. Our observations suggest that omalizumab represents a valuable therapeutic option in the management of BP patients. Its efficacy might be related to reduction in FcεRI+ and IgE+ basophils and intradermal cells.

## Introduction

Bullous pemphigoid (BP) is a blistering antibody-mediated autoimmune skin disease. BP is associated with tissue-bound and circulating autoantibodies directed against two hemidesmosomal proteins, BP180 (also called BPAG2, type XVII collagen) and/or BP230 (also called BPAG1-e) (1, 2). For many decades, studies examining the pathogenic mechanisms of BP autoantibodies focused solely on IgG (3). In recent years, presence of IgE antibodies against the basement membrane zone components, elevated serum IgE and BP180-specific IgE in BP sera suggested that IgE has a role in BP pathogenesis (1–14). As a result, humanized monoclonal antibody directed to IgE, omalizumab, an approved treatment for severe asthma and chronic spontaneous urticaria, might represent an alternative drug for BP. Although the clinical effectiveness of omalizumab in treatment of BP has been already demonstrated (1, 2, 12, 15–18), the exact mechanism of action remains elusive. Furthermore, current data suggest that only a subset of BP patients respond favorably to omalizumab treatment. However, biomarkers to identify the subset of BP that profits from anti-IgE treatment have yet to be identified (19). In chronic spontaneous urticaria, changes in serum IgE levels and in FcεRI expression on basophils post anti-IgE therapy have been linked to therapy success. We aimed therefore in this study to assess the effect of omalizumab on FcεRI and IgE expression on circulating basophils and on lesional intradermal cells in BP to generate insight into the immunological effects of omalizumab in BP.

## Report of the Cases

Two patients with severe recalcitrant BP treated with omalizumab at Department of Dermatology, Bern University Hospital were included after informed consent. Both patients have suffered from intense pruritus since long time. The previous standard local and systemic therapies could not improve their complaints.

Efficacy of treatment was assessed clinically 4 months after initiation of the therapy. Lesional and non-lesional skin biopsies where taken before and 4 weeks after initiation of omalizumab therapy. In addition, FcεRI expression on circulating cells and IgE levels in serum and in the skin samples, as well as anti-BP180 and -BP230 in serum, eosinophils and basophils counts in blood were assessed before and during treatment, as discussed before (20, 21).

**Figure 1:**
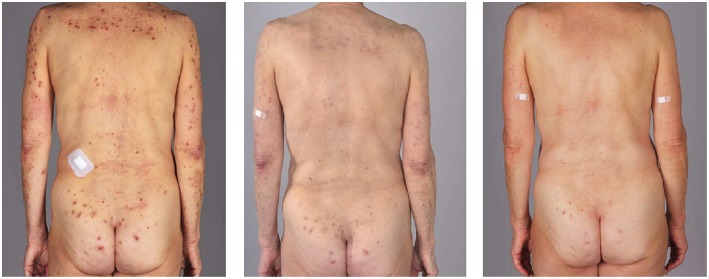
Clinical evaluation before (left), 10 days after (middle) and 4 months after the therapy in the first patient.

After 4 months of therapy (300 mg subcutaneously every 4 weeks), the patients were clinically disease-free and the pruritus subsided completely. The patients did not report any adverse events (Table 1, Figure 1).

**Table 1:** Patients characteristics.

**Patient no**.	**Age (range)**	**Diagnosis of BP**	**Previous Therapies**	**Before Therapy assessments**	**Therapy regime**	**After Therapy assessments**	**Clinical response**
1	60–65	- Intense pruritus, blisters since 2 years - Subepidermal blister in histological examination of the lesional skin - Linear deposits of IgG and complement C3 at the dermal-epidermal junction in direct immunofluorescence of lesional/perilesional skin	- Topical corticosteroid - Systemic corticosteroids - Azathioprine - Tetracycline/nicotinamide	- Serum level of IgE (1,176 U/L) and basophil membrane FcεRI (42′778) - Eosinophilia (0.62 G/L) - Circulating IgG antibodies against BP-180 (45 U/L) and BP-230 (106 U/L) - Normal basophil count	−300 mg subcutaneously every 2 weeks initially for the first month in combination with topical steroid and then in monthly regime in monotherapy	- Immunohistochemical analysis of the skin biopsy, showed a notable decrease in the tissue FcεRI^+^ and IgE^+^ cells after 4 weeks of the treatment - Decreased basophil-membrane FcεRI (3′632 after 1 month; 3′063 after 4 months of the treatment) - Serum total IgE during treatment showed an increase after 1 month to 1,843 U/L with further relative decrease after 4 months (1,466 U/L) and increase after 6 months (2202 U/L). - Circulating IgG antibodies against BP-180 (21.3 U/L) and BP-230 (97.3 U/L) after 6 months - Eosinophil count decreased after 4 weeks of treatment (0.22 G/L).	- After 4 months of therapy the patient's skin was clinically disease-free and pruritus subsided completely. - No adverse events were reported.
2	70–75	- A long history of itchy dermatosis without blisters - Subepidermal blister with an eosinophil-rich inflammation in histological examination of the lesional skin - Linear deposits of IgG, IgE and C3 at the dermal-epidermal junction in direct immunofluorescence of perilesional skin	- Topical corticosteroid - Systemic corticosteroids	- Serum level of IgE (410 U/L) and basophil membrane FcεRI (97′201). - Normal eosinophil count (0.34 G/L) - Circulating IgG antibodies against BP-180 (21.7 U/L) and BP-230 (11.5 U/L) - Normal basophil count	300 mg subcutaneously monthly, initially in combination with topical steroid and then in monotherapy	- Immunohistochemical analysis of the skin biopsy, showed a notable decrease in the tissue FcεRI^+^ and IgE^+^ cells after 4 weeks of the treatment - Decreased basophil-membrane FcεRI [3′678 after 1 month, remained low after 5 months of treatment (6′367)]. - Serum total IgE during treatment showed a slight increase after 1 month to 553 U/L with further values fluctuating between 650 and 730 U/L in the following months. - Circulating IgG antibodies against BP-180 (17.9 U/L) and BP-230 (7.3 U/L) after 6 months - Basophils granulocytes remained low during treatment - Eosinophil counts slightly increased (0.37 after 1 month, 0.83 after 4 months).	

**Figure 2:**
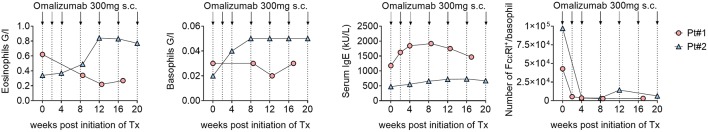
Despite therapy with omalizumab the eosinophils and basophils increased during therapy in the second patient. IgE level in serum did not also decrease. However, serum level of FcεRI decreased dramatically.

**Figure 3:**
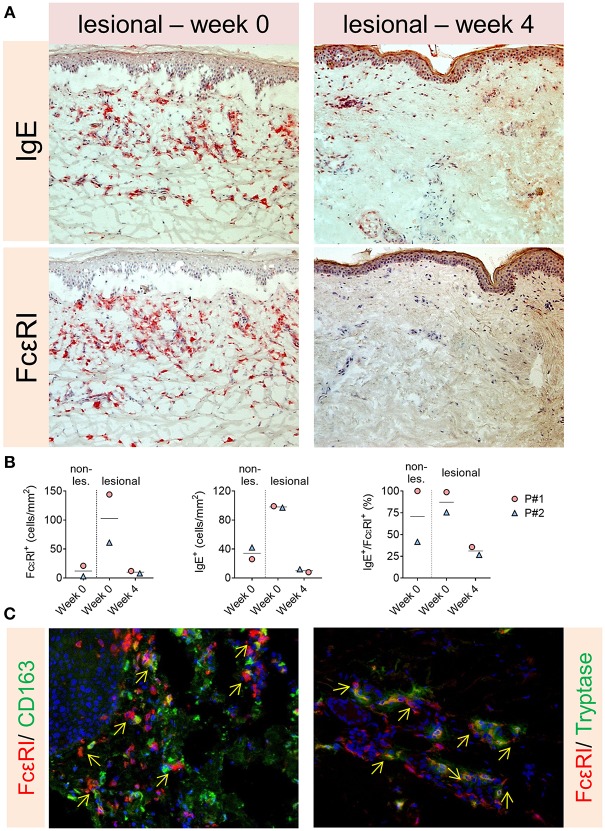
**(A,B)** Significant reduction of IgE^+^ and FcεRI^+^ cells in dermis in the immunohistochemistry stainings of the patients after 4 weeks of omalizumab treatment. **(C)** Both mast cells and APCs were found to express FcεRI in lesional skin (arrows show double positive cells).

The eosinophils, basophils and IgE level in serum were variably affected and differed between the two patients. The BP-180/230 level decreased also slightly in our patients under Therapy. However, levels of FcεRI on circulating basophils decreased dramatically. Furthermore, a significant reduction of IgE+ and FcεRI+ cells in dermis were observed in the immunohistochemistry stainings after 4 weeks of omalizumab treatment (Figures 2, 3). To investigate the cellular source of FcεRI-carrying cells, we performed double immunofluorescence in lesional skin for markers of mast cells (tryptase) and antigen presenting cells (CD163) together with FcεRI staining. Both mast cells and APCs were found to express FcεRI in lesional skin (Figure 3).

## Discussion

Our study in two patients with therapy-resistant BP in which omalizumab was used as rescue therapy has assessed the impact of omalizumab on levels of eosinophils, basophils, and IgE in the circulation, on FcεRI expression on circulating basophils, and on lesional expression of IgE and FcεRI in skin biopsy samples. Our observations suggest that changes in lesional IgE and FcεRI expression might be useful to distinguish the subset of BP patients who benefit from omalizumab therapy.

Frequent detection of high levels of IgE autoantibodies against BP180 and BP230, increased serum levels of IgE and the reported beneficial effect of anti-IgE therapy in small case series support the idea that IgE autoantibodies are involved in BP pathogenesis (1, 2, 5, 10, 12, 15–18, 22–24). However, the pathogenicity of IgE autoantibody in BP remains unclear (25). It is thought that IgE autoantibodies directed against the extracellular domain of BP180 are first bound to FcεRI on mast cells and eosinophils. This binding subsequently promotes degranulation and initiates an inflammatory reaction resulting in further tissue damage and blister formation (2, 12, 26). In addition, binding of specific IgE autoantibodies to the ectodomain of BP180 on basal keratinocytes may lead to internalization of BP180 and thereby contribute to cell-substrate dysadesion and blister formation (2, 12, 16).

Recent studies found that high serum levels of IgE correlate with disease severity in BP. In our two cases, however, there was no reduction of serum IgE levels 4 months after omalizumab therapy compared to baseline, despite clinical remission. It is conceivable that omalizumab, although it did not reduce the total IgE serum levels in our patients, is able to sequester free IgE and prevent its binding to its high-affinity IgE receptor, FcεRI (7, 27, 28). This process has been proposed to then down-regulate the expression of FcεRI on mast cells and basophils as well as antigen-presenting cells (7). In addition to its ability to neutralize the free IgE, omalizumab causes dissociation of IgE from the IgE-FcεRI complex (27, 28). IgE-free FcεRI is unstable and is then internalized for degradation. By this means, the activation of mast cells and basophils is reduced (7, 27–29). In line with these hypotheses, we found a sharp decrease of FcεRI expression on circulating basophils and a strong reduction of FcεRI+ cells in the skin of both patients after 4 weeks of omalizumab treatment (Figures 2, 3). These events might consequently lead to local depletion of IgE, as reflected by a strong reduction of IgE+ cells in dermis of both our patients after 4 weeks of omalizumab treatment. Interestingly, Metz et al. showed in a similar study that clinical efficacy of omalizumab in chronic spontaneous urticaria is associated with decreases in FcεRI^+^ cells and IgE^+^ cells in lesional and non-lesional skin (dermis) of patients (to levels seen in healthy subjects) (30).

BP180 and BP230 ELISAs are highly sensitive methods for the diagnosis of BP, in particular BP180 ELISA, is a sensitive tool for monitoring disease activity (31–33). However, in our patients no decrease in the level of specific IgG BP180 and BP230 autoantibodies was observed after 4 months, despite marked clinical improvement. In line with this observation, Balakirski et al. reported no changes in circulating autoantibodies within 6 months after the treatment with omalizumab (16). Similarly, Yu et al. noticed only gradual decline in the level of these antibodies in 6 BP patients treated with omalizumab (23). It is thus possible that in a subset of BP patients, disease activity is not entirely driven by BP180/230-specific IgG antibodies but that IgE autoantibodies also substantially contribute to disease. It is tempting to speculate that these BP patients might represent the subset most likely to respond to anti-IgE treatment. Further studies are needed to investigate this hypothesis. Peripheral eosinophilia has been reported in 50–70% of BP patients (3, 22, 34–37). de Graauw et al. demonstrated that IL-5-activated eosinophils directly contribute to BP blister formation in the presence of BP autoantibodies. Dermal–epidermal separation by IL-5-activated eosinophils depends on adhesion and Fcγ receptor activation, requires elevated reactive oxygen species production, degranulation and involves eosinophil extracellular trap formation (38). It has recently been shown that peripheral eosinophilia correlated with the overall disease activity and the extent of erosions and blisters (3). Interestingly, our second patient did not have an elevated eosinophil count before treatment but showed rapid clinical improvement suggesting that peripheral eosinophilia might not serve as biomarker for response to omalizumab. In contrast, it has been claimed that asthma patients with a higher baseline peripheral eosinophil count show a better clinical outcome after omalizumab (39). To what extent the mechanisms of action of omalizumab is shared between BP, allergic asthma, and chronic spontaneous urticaria remains to be elucidated.

Our findings support the idea that IgE autoantibodies contribute to tissue damage. Prospective, randomized controlled trials are necessary to confirm that omalizumab represents a new treatment option for BP. However, the current literature suggests that only a subset of patients responds to omalizumab therapy. Therefore, it is paramount to identify biomarkers that predict the best treatment for the individual BP patient. Biomarker development typically relies on a solid understanding of the pathomechanisms at play. In this context, the exact mechanisms by which IgE contributes to pathogenesis in BP is not completely clear. Our data now suggests that optimal clinical responses to anti-IgE treatment in BP might be associated with dissociation of the IgE-FcεRI complex, consecutive down-regulation of FcεRI expression, and finally a decrease in lesional IgE-bearing immune cells. These observations provide novel insight into the immunomodulatory effects at the tissue level of anti-IgE treatment in BP and lay an important basis for future studies.

## Data Availability

The raw data supporting the conclusions of this manuscript will be made available by the authors, without undue reservation, to any qualified researcher.

## Ethics Statement

Patients gave written informed consent for research use of their data.

## Author Contributions

SS, KG, LF, LB, NY, and CS designed the study and performed acquisition, analysis, interpretation of data, and critical revision of the manuscript for important intellectual content. SS, KG, and CS wrote the manuscript.

### Conflict of Interest Statement

NY has received honoraria for consulting and advisory board attendance from Novartis. The remaining authors declare that the research was conducted in the absence of any commercial or financial relationships that could be construed as a potential conflict of interest.
